# Dietary and drinking water intake of essential trace elements in a typical Kashin-Beck disease endemic area of Tibet, China

**DOI:** 10.1186/s12940-022-00898-0

**Published:** 2022-09-16

**Authors:** Xinjie Zha, Jialu An, Xue Gao, Yuan Tian

**Affiliations:** 1 Xi’an University of Finance and Economics, Changning Str. 360, Chang’an District, Xi’an, 710100 China; 2 Institute of Agricultural Resources and Environment, Tibet Academy of Agriculture and Animal Husbandry Sciences, Jinzhu Str.130, Chengguan District, Lhasa, 850000 China; 3 Key Laboratory of Ecosystem Network Observation and Modelling, Institute of Geographic Sciences and Natural Resources Research, Chinese Academy of Sciences, Datun Str. 11A, Chaoyang District, Beijing, 100101 China

**Keywords:** Essential trace elements, Highland barley, Drinking-water, Oral intake, Health risk assessment

## Abstract

**Background:**

Essential trace elements (ETEs), such as copper (Cu), iron (Fe), manganese (Mn), molybdenum (Mo), selenium (Se), zinc (Zn), are very important elements for human health.

**Methods:**

In this study, 89 drinking water samples and 85 highland barleys were collected from 48 villages in 11 townships, and the average daily dose (*ADD*) of ETEs were calculated, in addition, health effects of ETEs to rural residents in Luolong County, a typical Kashin-Beck disease (KBD) endemic area in Tibet, were assessed.

**Results:**

The mean concentrations of Cu, Fe, Mn, Mo, Se, Zn in drinking water were 0.278 ± 0.264 μg·kg^−1^, 0.766 ± 0.312 μg·kg^−1^, 0.411 ± 0.526 μg·kg^−1^, 0.119 ± 0.223 μg·kg^−1^, 0.155 ± 0.180 μg·kg^−1^, and 0.804 ± 1.112 μg·kg^−1^, respectively; and mean concentrations of Cu, Fe, Mn, Mo, Se and Zn in highland barley were 3.550 ± 0.680 mg·kg^−1^, 81.17 ± 38.14 mg·kg^−1^, 14.03 ± 1.42 mg·kg^−1^, 0.350 ± 0.200 mg·kg^−1^, 0.0028 ± 0.0056 mg·kg^−1^, and 23.58 ± 3.10 mg·kg^−1^, respectively. The *ADD* of Cu in the study area was appropriate; the *ADD* of Fe and Mn in each township were higher than the maximum oral reference dose recommended by the National Health Commission of China, indicating that Fe and Mn had non-carcinogenic health risks; the *ADD* of Mo and Zn in 36.36% and 54.55% of the townships exceeded the maximum oral reference dose; and 72.73% of the townships had insufficient *ADD* of Se. The *ADD* of Mo, Cu and Se in different townships was significantly correlated with the prevalence of KBD.

**Conclusions:**

Therefore, in order to prevent and control the prevalence of KBD and ensure the health of local residents, it is necessary to reduce the intake of high concentrations of Fe, Mn and Zn in diet, as well as increase the intake of Mo, Cu, especially Se.

**Graphical abstract:**

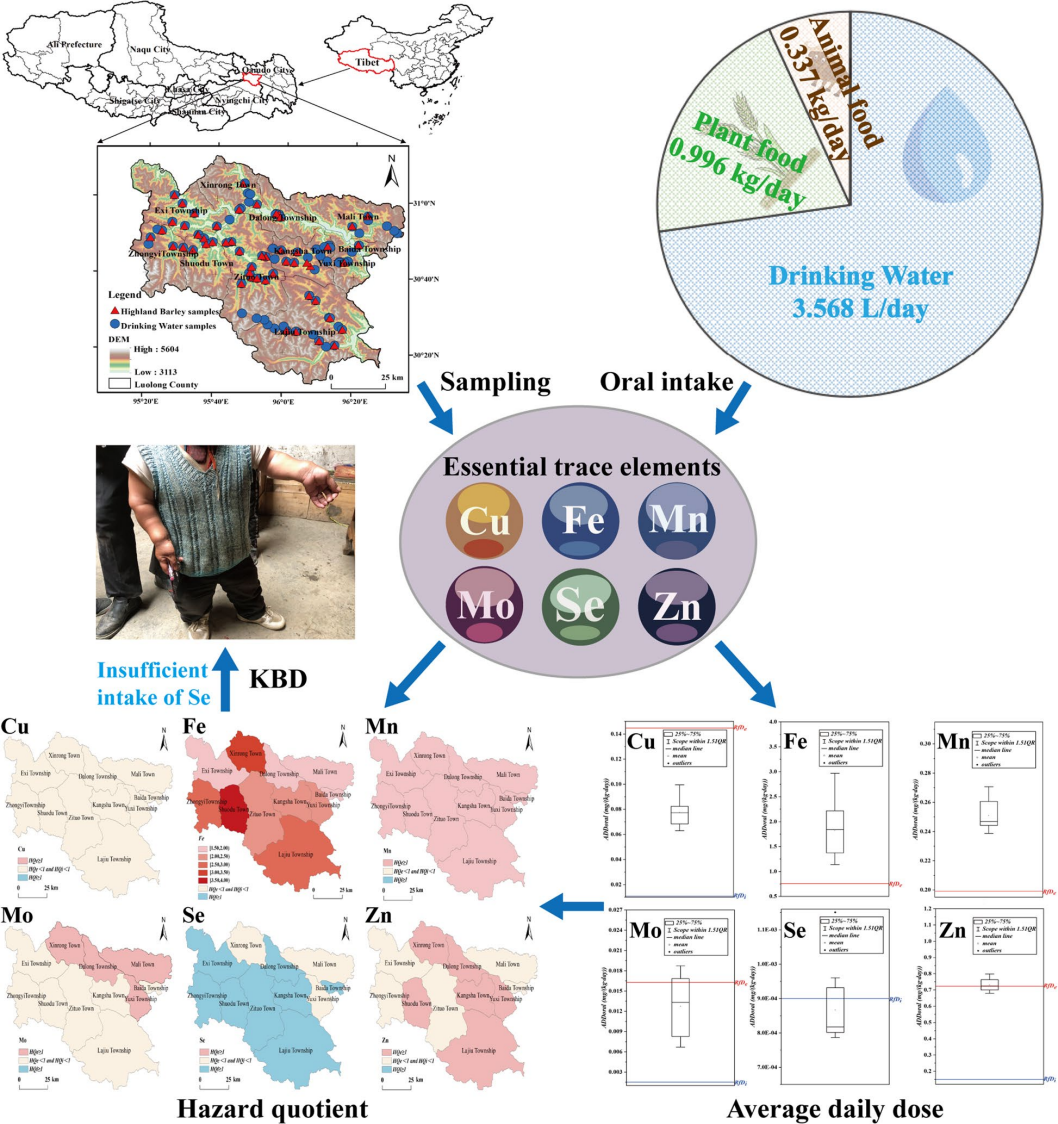

**Supplementary Information:**

The online version contains supplementary material available at 10.1186/s12940-022-00898-0.

## Introduction

Kashin–Beck Disease (KBD) is a chronic, degenerative, endemic osteochondropathy involving growth cartilage and plates [1, 2]. KBD has essentially controlled in China at present through a series of measures such as rural drinking water safety project, grain replacement, and relocation during the 12th and 13th Five-Year Plans period, however, Qamdo City is the most serious and active area of KBD currently [3–6]. Although a number of studies have been conducted on the etiology of KBD, the most studied and best established KBD etiological theory by researchers have increasingly tended toward concluding that endemic deficiency of trace elements in local diets [7, 8]. Existing studies generally believe that biogeochemical selenium (Se) deficiency is the main cause of KBD [9, 10], while some other essential trace elements (ETEs) such as copper (Cu), iron (Fe), manganese (Mn), molybdenum (Mo) and zinc (Zn) also have certain biological functions and play an important role in the prevalence of KBD [2, 5, 10–14].

With the continuous change of environment caused by human activities, the impact of the hypergene environment on human health through the biogeochemical cycle of trace elements has also received widespread attention [15–17]. In natural ecosystems, grain and water are the most common ecosystem supply services, and oral intake of grain and water is also the most important way for people to intake ETEs [18, 19]. However, due to the narrow thresholds of ETEs, long-term insufficient or excessive dietary intake can lead to health risks and potential toxicity [20–22]. Therefore, ETEs in diet and drinking water have important impacts on human health and life quality [23, 24]. The United States Environmental Protection Agency (USEPA), the World Health Organization (WHO), and the European Food Safety Authority (EFSA) all take health risk assessment as an effective way to improve drinking water and food safety, and reduce dietary intake-related diseases [25–28]. China has also formulated recommended intake standards for ETEs in drinking water and food, and guidelines for health risk assessment [29, 30].

The Qinghai-Tibet Plateau is the most unique physiographic unit in the world, which has obvious regional environmental effects on the health of local residents [31, 32]. As a result, the Qinghai-Tibet Plateau is China’s most serious and active epidemic area [33], especially KBD [4, 9]. Huang et al. [34] found that diet is the primary source of trace elements for rural residents in Qinghai-Tibet Plateau. Hence, calculating the local residents’ ETEs intake and assessing their health effects are crucial. Zha et al. [35] sampled and analyzed the concentration of Se in surface water of Tibet, and revealed that the Se concentration in Tibet’s surface water was relatively low, with an average value of 0.519 μg/L (range of 0–3.944 μg/L). Tian et al. [36] studied the concentrations of Cu and Mo of natural pasture plants and surface water in northern Tibet, and found that there were obvious regional differences in Cu and Mo concentrations, but there was no risk to human health. Zha et al. [2] and Shi et al. [10] found that there were significant differences of concentrations of some ETEs such as Se, Fe Zn and Mo in drinking natural water between KBD endemic areas and non-endemic areas in the Qinghai-Tibet Plateau. In addition, the existing studies on the intake of trace elements in the Qinghai-Tibet Plateau mainly focused on some trace elements in endemic areas, such as Se in the KBD endemic areas [8, 37, 38] and arsenic in arsenic poisoning endemic areas [39, 40]. According to our review of the literature, there are few systematic studies on the dietary and drinking water intake of multiple ETEs in the KBD endemic area of the Qinghai-Tibet Plateau.

Based on the above considerations, this paper takes Luolong County as the study area, systematically collects and analyzes drinking water and highland barley samples from 48 administrative villages (72.73% of the total administrative villages) in all 11 townships of Luolong County, and attempts to: (a) identify the concentration and distribution of ETEs in highland barley and drinking water; (b) calculate the ADD of ETEs in diet and drinking water of residents in each township; (c) assess the health effects of ETEs on residents and explore its correlation with the prevalence of KBD. The results of this study can be helpful to quantitatively evaluate the health effects of ETEs intake in residents’ daily diet, which can be conducive to providing scientific basis for the prevention and control of KBD in the study area.

## Materials and methods

### Study area description

**Fig. 1:**
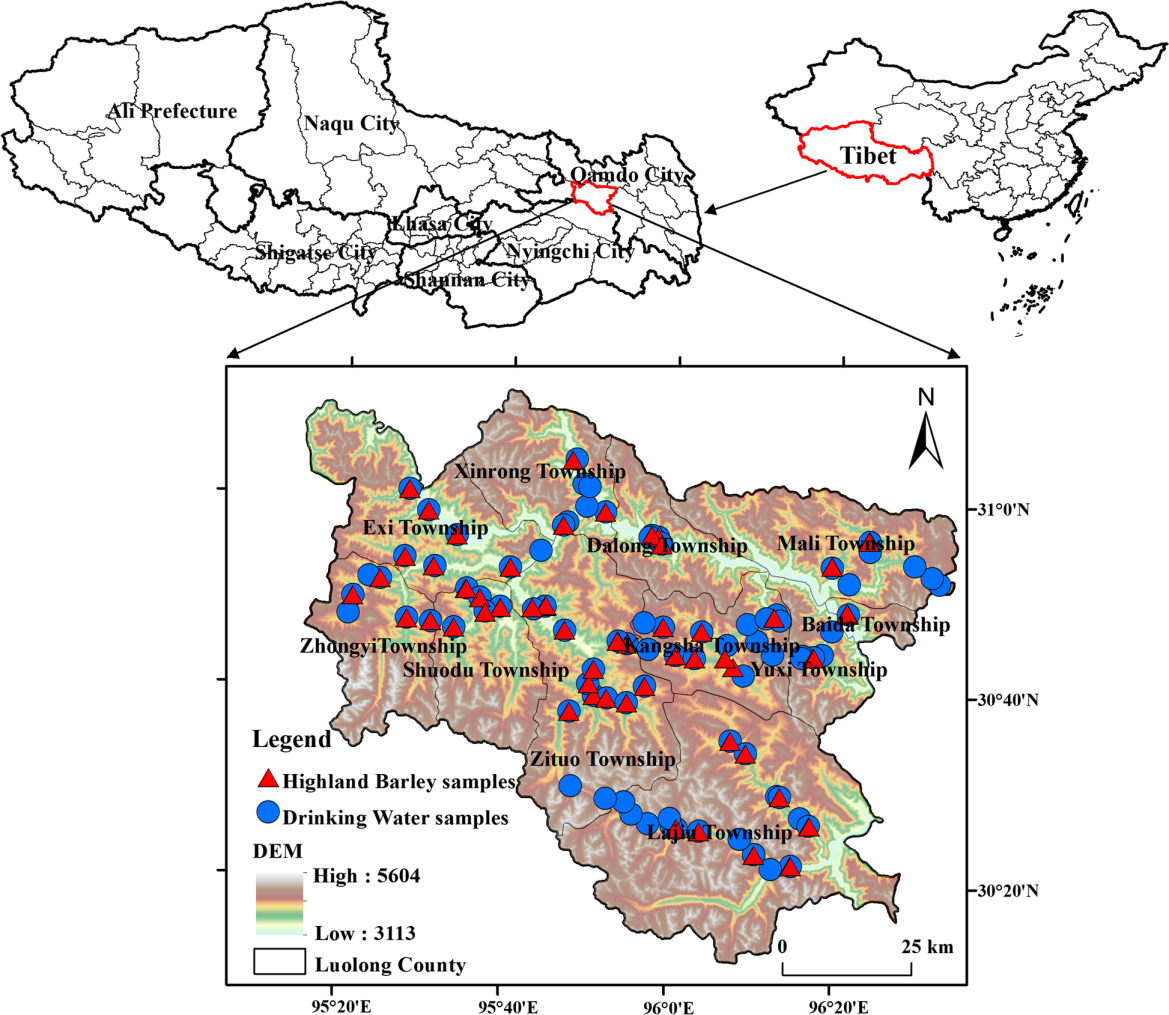
Distribution of sampling sites

Luolong County (95°10′–95°50′ E, 30°10′–31°50′ N), is located in the northwest of Hengduan Mountains in northeastern Tibet and the middle reaches of Nujiang River (Fig. 1). Luolong County has a total of 11 townships with an area of about 8108 km^2^, and there are about 53,185 people in the county according to the seventh population census data. The annual precipitation in Luolong County is about 423.7 mm, and the terrain is dominated by alpine canyons, with an average altitude of about 4338.14 m. According to the epidemiological survey data of KBD provided by Luolong County Health Commission, the detection rate of KBD in the whole county is 16.18%, which is one of the most seriously epidemic counties in Tibet.

### Sampling and preparation

In this study, 89 drinking water samples and 85 highland barley samples were collected from 11 townships of Luolong in August 2021. The Global Position System (GPS) coordinates of sampling sites were recorded by handheld GPS devices (Etrex 221x, GARMIN, Taiwan, China). Samples were collected and pretreated on basis of the following methods:

- Drinking natural water samples were collected and stored in 300 mL colorless polyethylene bottles that had been pre-rinsed with deionized water, and kept at 4 degrees centigrade (℃) during storage and transportation. Water samples were collected and then stored according to China’s Standard Examination Methods for Drinking Water (GB/T 5750–2006).
- Each sample collected about 200 g of food samplesand then stored in the colorless polyethylene plastic bags. During pretreatment process in the Laboratory of Lhasa Agricultural Ecological Experiment Station, Chinese Academy of Sciences (CAS), all food samples were washed with deionized water and then dried in an oven at 60 °C for at least 48 h. Then, the post-drying food samples were grinded to less than 200 mesh with an agate mill.

### Sample analysis

Samples were tested using the following methods at the Laboratory of Analytical and Testing Center, Institute of Geographic Sciences and Natural Resources Research, CAS:

- Drinking water samples: the concentration of Cu, Fe, Mo, Mn, Zn were tested by the Inductively Coupled Plasma Mass Spectrometry (ICP–MS, DRC-e, PerkinElmer, Massachusetts State, USA, LOD: 0.001 µg/L). Se contents were determined by Hydride Generation Atomic Fluorescence Spectrometry (HG–AFS, AFS-9780, Haiguang, Beijing, China, LOD: 0.01 µg/L).
- Food samples: About 0.5 g of each food sample was taken for analysis. The mixed acid of HNO_3_ and HClO_4_ (5:1) was added and heated at 180 ± 10 °C until the solution was transparent, and the major and trace elements were determined by ICP–MS. The mixed acid of HNO_3_ and HClO_4_ (9:1) was added, and heated at 180 ± 10 °C until it was transparent. Then, 5 mL of 6 mol/L HCl was added after cooling, and the digestion was allowed to continue until white smoke was formed. Then 1 mL HCl was added, and the Se concentration was determined by HG–AFS.

In the analysis process, quality assurance was achieved by using certified external National Standard Substances and repeated measurements (repeated sample was tested after every 10 samples): (1) Cationic external standard solutions for drinking water samples were prepared with the Multi-element ICP–MS Calibration Standards (Lot# 15-76JB, Cat# N9300233); (2) National Standard Substances (GB, provided by the Certified Reference Material Center, China) for food were prepared from GBW10011 (wheat), GBW10015 (spinach) and GBW10044 (rice in Sichuan Province), respectively. The relative error (equivalent ratio) of each GB sample was less than 5.00% and the correlation coefficient was greater than 0.999. Therefore, it can be said that the analysis methods and data of this study are reliable and accurate.

### Estimated daily intake

The main source of carbohydrates for residents in the study area is local highland barley [41]. It is generally believed that the main route of exposure to trace elements is direct intake from the diet (drinking water and highland barley) [42]. In this study, the average daily dose and health risk were evaluated based on the method given in the Technical Guidelines for Environmental Health Risk Assessment of Chemicals in China [29]. To calculate both the addition of food consumption and drinking water, the formula is as follows:

1 $${ADD}_{w}=\frac{{C}_{w}\times {IR}_{w}\times EF\times ED}{BW\times AT}$$

2 $${ADD}_{h}=\frac{{C}_{h}\times {IR}_{h}\times EF\times ED}{BW\times AT}$$

3 $${ADD}_{m}=\frac{{C}_{m}\times {IR}_{m}\times EF\times ED}{BW\times AT}$$

4 $${ADD}_{oral}={ADD}_{w}+{ADD}_{h}+{ADD}_{m}$$

where *ADD* means average daily dose (unit: mg/(kg·day)), *C*_*w*_, *C*_*h*_ and *C*_*m*_ is the mean concentration of trace elements in drinking water (unit: mg/L), plant food (unit: mg/kg) and animal food (unit: mg/kg), respectively. *IR* represents the ingestion rate. The values of *IR*_*h*_ and *IR*_*m*_ are calculated according to the data of participatory rural assessment (PRA) in Tibet (*n* = 231) by Wang et al. [43], and the value of *IR*_*w*_ is calculated according to the PRA data in Tibet (*n* = 1143) by MEP [44]. For adults, the values of drinking water, plant food and animal food are 3.568 L/day, 0.996 kg/day and 0.337 kg/day, respectively. EF represents the exposure frequency of 365 days/year for each group (in days/year). ED indicates an exposure time of 45 years. BW represents a body weight of 55.3 kg. AT represents the average exposure time of 16,425 days.

### Human health risk assessment

According to the National Health Commission of the People’s Republic of China (2021) [29], the non-carcinogenic risk can be defined by the hazard quotient (*HQ*), which is calculated by Eqs. (5) and (6).

5 $${HQ}_{e}=\frac{{ADD}_{oral}}{{RfD}_{e}}$$

6 $${HQ}_{i}=\frac{{RfD}_{i}}{{ADD}_{oral}}$$

where *RfD* (reference dose) is the exposure dose for non-carcinogenic risk, also known as the reference dose, and the unit is mg/(kg·day). Reference doses for different elements are shown in Table 1. *HQ* represents the ratio of the daily average exposure dose of non-carcinogenic substances to the reference value, which is dimensionless, indicating the non-carcinogenic risk of elements; When *HQ* is greater than 1, there might be a non-carcinogenic effect. The subscripts “*e*” and “*i*” represent “excessive” and “insufficient” respectively.

**Table 1:** Reference doses of different elements (*RfD*) (unit: mg/(kg·day))

ETEs	Cu	Fe	Mn	Mo	Se	Zn	Reference
*RfD*_*i*_	0.0108	0.2170	0.0743	0.0015	0.0009	0.1492	[29, 45]
*RfD*_*e*_	0.1447	0.7595	0.1989	0.0163	0.0072	0.7233	

## Results

The average concentrations of Cu, Fe, Mn, Mo, Se and Zn in drinking water samples of each township in Luolong County (Table 2), were far lower than the limits given by the Standards for Drinking Water Quality (GB5749–2022) [46].

**Table 2:** Mean concentrations of ETEs in drinking water samples (unit: μg/L)

Survey locations	Cu	Fe	Mn	Mo	Se	Zn
Baida Township (*n* = 1)	0.189	0.370	0.041	1.252	0.354	0.665
Dalong Township (*n* = 5)	0.256 ± 0.100	0.897 ± 0.222	0.040 ± 0.025	1.693 ± 1.636	0.198 ± 0.138	0.919 ± 0.701
Exi Township (*n* = 7)	0.183 ± 0.172	0.706 ± 0.368	0.038 ± 0.017	0.088 ± 0.008	0.088 ± 0.067	0.614 ± 0.290
Kangsha Township (*n* = 14)	0.200 ± 0.176	0.514 ± 0.744	0.091 ± 0.107	0.175 ± 0.164	0.085 ± 0.077	0.468 ± 0.246
Najiu Township (*n* = 20)	0.128 ± 0.091	0.688 ± 0.647	0.034 ± 0.023	0.652 ± 0.628	0.035 ± 0.023	0.392 ± 0.196
Mali Township (*n* = 7)	0.206 ± 0.100	0.384 ± 0.206	0.054 ± 0.024	0.432 ± 0.354	0.303 ± 0.130	0.932 ± 0.961
Shuodu Township (*n* = 5)	2.672 ± 2.239	0.264 ± 0.139	1.370 ± 2.288	0.219 ± 0.286	0.074 ± 0.066	1.965 ± 2.135
Xinrong Township (*n* = 7)	0.198 ± 0.146	0.963 ± 0.825	0.075 ± 0.071	0.105 ± 0.004	0.336 ± 0.295	0.850 ± 0.431
Yuxi Township (*n* = 5)	0.141 ± 0.074	0.215 ± 0.088	0.058 ± 0.050	0.273 ± 0.070	0.308 ± 0.146	0.560 ± 0.501
Zhongyi Township (*n* = 6)	0.747 ± 0.279	1.070 ± 0.287	0.452 ± 0.591	0.083 ± 0.078	0.149 ± 0.207	2.114 ± 2.780
Zituo Township (*n* = 12)	0.270 ± 0.279	4.871 ± 8.230	0.075 ± 0.088	0.491 ± 0.658	0.066 ± 0.049	3.311 ± 4.777
Luolong County (*n* = 89)	0.278 ± 0.264	0.766 ± 0.312	0.411 ± 0.526	0.119 ± 0.223	0.155 ± 0.180	0.804 ± 1.112
GB 5749–2022	1000	300	100	70	10	1000

The average concentrations of Cu, Fe, Mn, Mo, Se and Zn in highland barley from different townships in Luolong County were listed in Table 3. Mean concentrations of Cu, Fe and Zn in highland barley in Tibet were 4.94 mg/kg, 127.83 mg/kg and 49.13 mg/kg, respectively [47, 48]. It can be seen from Table 3 that the concentrations of Fe, Cu and Zn in highland barley in Luolong County were lower than the average value in Tibet. In view of the National Food Safety Standards for Pollutant Limits in Food (GB 2762–2017) and Food Safety National Standard of Grain (GB 2715–2016) [29, 49], the concentrations of Cu and Zn in highland barley in each township did not exceed the limits of these standards. In addition, the concentration of Fe exceeded the limit value, and the excess multiples ranged from 1.02 times (Dalong) to 2.78 times (Shuodu), and the Se concentration was far below the limit value. These data revealed that highland barley in Luolong County had high Fe content and low Se content.

**Table 3:** Mean concentration of ETEs in highland barley samples (mg/kg)

Survey locations	Cu	Fe	Mn	Mo	Se	Zn
Baida Township (*n* = 2)	3.879 ± 0.266	37.830 ± 3.564	13.195 ± 0.233	0.509 ± 0.001	0.005 ± 0.002	21.205 ± 2.355
Dalong Township (*n* = 3)	4.251 ± 1.081	51.100 ± 16.190	13.487 ± 0.337	0.728 ± 0.352	0.002 ± 0.002	27.127 ± 1.947
Exi Township (*n* = 8)	3.896 ± 0.732	52.531 ± 13.235	13.752 ± 0.687	0.217 ± 0.134	0.002 ± 0.001	22.913 ± 2.895
Kangsha Township (*n* = 9)	4.166 ± 0.649	76.837 ± 25.168	13.548 ± 1.358	0.615 ± 0.371	0.003 ± 0.001	25.862 ± 3.973
Najiu Township (*n* = 15)	3.424 ± 0.542	84.864 ± 25.234	14.099 ± 1.659	0.404 ± 0.212	0.002 ± 0.001	23.741 ± 2.950
Mali Township (*n* = 6)	4.443 ± 0.835	58.553 ± 34.904	13.988 ± 1.826	0.729 ± 0.244	0.010 ± 0.004	22.345 ± 5.951
Shuodu Township (*n* = 15)	3.129 ± 0.510	139.636 ± 70.124	14.966 ± 1.003	0.228 ± 0.109	0.002 ± 0.001	24.630 ± 1.937
Xinrong Township (*n* = 3)	3.658 ± 0.480	107.737 ± 47.750	13.563 ± 0.388	0.566 ± 0.255	0.009 ± 0.011	23.823 ± 1.839
Yuxi Township (*n* = 2)	4.735 ± 0.761	60.480 ± 2.560	14.440 ± 2.843	0.661 ± 0.082	0.021 ± 0.011	27.825 ± 8.365
Zhongyi Township (*n* = 4)	3.223 ± 0.503	97.742 ± 33.756	13.497 ± 1.957	0.353 ± 0.066	0.003 ± 0.001	23.605 ± 3.362
Zituo Township (*n* = 18)	3.223 ± 0.454	79.218 ± 42.378	13.644 ± 1.301	0.229 ± 0.120	0.001 ± 0.001	21.831 ± 1.210
Luolong County (*n* = 85)	3.550 ± 0.677	81.170 ± 38.143	14.030 ± 1.419	0.350 ± 0.198	0.003 ± 0.006	23.580 ± 3.103
GB 2762–2017/GB 2715–2016	10	50	–	–	0.3	50

The average concentration of ETEs in animal foods was based on the data of yak meat in Qamdo City measured by Wu [50] and Wu [51]. Cu (*n* = 15) was 1.362 mg/kg, Fe (*n* = 15) was 74.71 mg/kg, Mn (*n* = 15) was 0.178 mg/kg, Mo (*n* = 8) was 0.417 mg/kg, Se (*n* = 15) was 0.125 mg/kg and Zn (*n* = 15) was 48.82 mg/kg. The *ADD* of ETEs in drinking water, highland barley and meat were calculated according to Eqs. (1), (2) and (3), and the results are shown in the appendix (Table S3, S4 and S5). The *ADD* values of Mn, Fe, Cu and Zn intake through drinking water in Southwest region were higher than those in other regions, mainly in Shuodu, Zituo and Zhongyi Townships. The *ADD* of Mo was higher in the northeast region, mainly distributed in townships such as Dalong, Baida and Mali, while the *ADD* of Se was low in all townships. The *ADD* values of Fe and Mn in highland barley were the highest in Shuodu Township; the *ADDs* of Cu and Zn were higher in the northeast region, mainly distributed in Yuxi, Mali and Baida Townships; The *ADD* value of Mo was the highest in Dalong Township, the *ADD* of Se was low in all townships. The intake of Fe was the highest ETEs in drinking water, highland barley and meat, whereas the intake of Se was the lowest in all these pathways. In addition, the ETEs intake of drinking water, highland barley and meat was in the order of highland barley > meat > drinking water. This finding was closely related to the daily production and living habits of the residents in the study area.

**Fig. 2:**
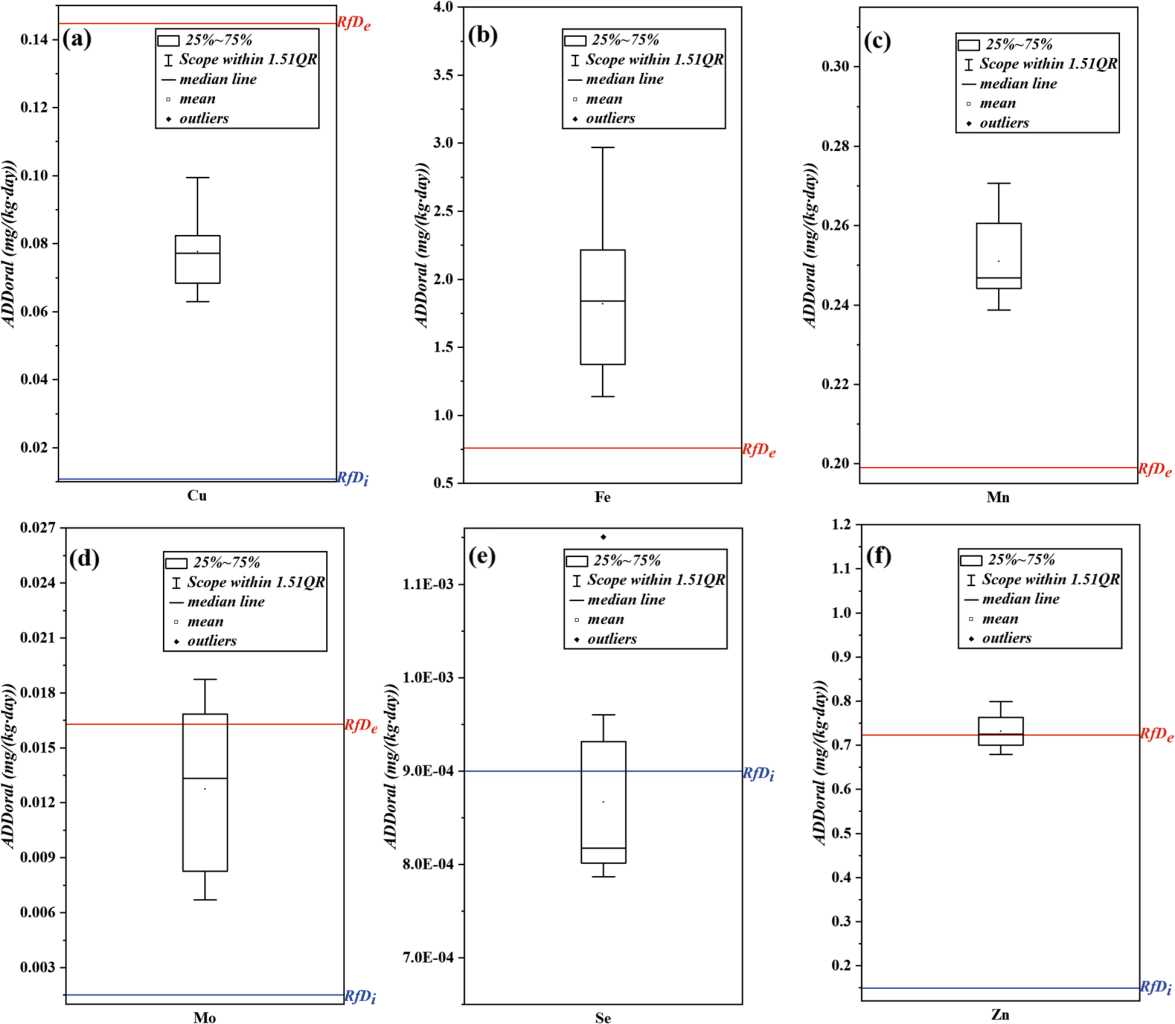
The *ADD*_*oral*_ and *RfD* of ETEs in Luolong County, **a** Cu, **b** Fe, **c** Mn, **d** Mo, **e** Se and **f** Zn

According to Eq. (4), the average daily oral dose (*ADD*_*oral*_) of ETEs of the study area was calculated (Table S6), and the relationship between the value of *ADD*_*oral*_ and the *RfD* in the whole study area were shown in Fig. 2. The order of *ADD*_*oral*_ of the six selected elements was Fe > Zn > Mn > Cu > Mo > Se, and the *ADD*_*oral*_ of Se in each township was low, which could be preliminarily inferred that Se intake in daily diet of the local residents was insufficient.

The *ADD*_*oral*_ of Cu, Mo and Se in different townships had a similar trend that the northeast is higher than the southwest in Luolong County, while the *ADD*_*oral*_ of Fe, Mn and Zn at township level (Fig. S1 and Table S6) hads no specific distribution trend. The *ADD*_*oral*_ values of Cu, was from 0.063 to 0.099 mg/(kg·day), which were higher than *RfD*_*i*_ and lower than *RfD*_*e*_, indicating that the amount of oral intake of Cu by residents in Luolong County is appropriate (Fig. 2a); The *ADD*_*oral*_ values of Fe, were from 1.137 to 2.970 mg/(kg·day), which were 1.497 to 3.911 times that of *RfD*_*e*_, indicating that the residents in Luolong County had excessive oral intake of Fe (Fig. 2b); The *ADD*_*oral*_ values of Mn, were from 0.239 to 0.271 mg/(kg·day), slightly higher than *RfD*_*e*_, indicating that the local residents’ oral intake of Mn is slightly excessive (Fig. 2c); The *ADD*_*oral*_ values of Mo, were from 0.0067 to 0.0189 mg/(kg·day), which were higher than *RfD*_*i*_ and most of them were lower than *RfD*_*e*_, indicating that the oral intake of Mo of residents is more appropriate (Fig. 2d); The *ADD*_*oral*_ values of Se, were from 0.00079 to 0.00115 mg/(kg·day), most of which were lower than *RfD*_*i*_, indicating that the residents in the study area have insufficient oral intake of Se (Fig. 2e); The *ADD*_*oral*_ values of Zn, were from 0.68 to 0.80 mg/(kg·day), which were higher than *RfD*_*i*_ and more than half of *RfD*_*e*_, indicating that the residents in the study area had a slightly excessive oral intake intake of Zn (Fig. 2f). In summary, there are significant spatial differences in the intake of these six ETEs, which is closely related to the geographical environment, geological background and human activities in the study area.

Based on the epidemiological survey data of KBD provided by the Health Commission of Luolong County (Table 4), the detection rate of KBD in each township was calculated and visualized (Fig. S2). According to the *Spearman’s* rank correlation coefficient, the *ADD*_*oral*_ of Mo, Cu and Se was significant negatively correlated (*p* < 0.05) with the prevalence of KBD at the township level in Luolong County, respectively (Table 4).

**Table 4:** Correlation analysis between Kashin-Beck disease and *ADD*_*oral*_ of ETEs

Survey locations	Mn	Fe	Cu	Zn	Se	Mo	KBD prevalence
Baida Township	0.2387	1.1367	0.0810	0.6795	0.0009	0.0133	7.33%
Dalong Township	0.2440	1.3757	0.0823	0.7861	0.0008	0.0187	2.95%
Exi Township	0.2488	1.4014	0.0772	0.7102	0.0008	0.0079	20.13%
Kangsha Township	0.2451	1.8392	0.0808	0.7633	0.0008	0.0138	8.01%
Lajiu Township	0.2550	1.9838	0.0629	0.7251	0.0008	0.0118	8.88%
Mali Township	0.2605	1.2579	0.0886	0.7000	0.0010	0.0167	3.49%
Shuodu Township	0.2707	2.9703	0.0684	0.7412	0.0008	0.0067	39.40%
Xinrong Township	0.2454	2.3958	0.0760	0.7266	0.0009	0.0172	6.80%
Yuxi Township	0.2612	1.5446	0.0994	0.7987	0.0012	0.0168	3.03%
Zhongyi Township	0.2442	2.2157	0.0693	0.7227	0.0008	0.0088	26.24%
Zituo Township	0.2468	1.8823	0.0681	0.6908	0.0008	0.0083	16.78%
*Spearman’s* rank correlation coefficient	0.182	0.573	–0.755^**^	–0.327	–0.628^*^	–0.936^**^	–

^*^means *p* < 0.05

^**^means *p* < 0.01

**Fig. 3:**
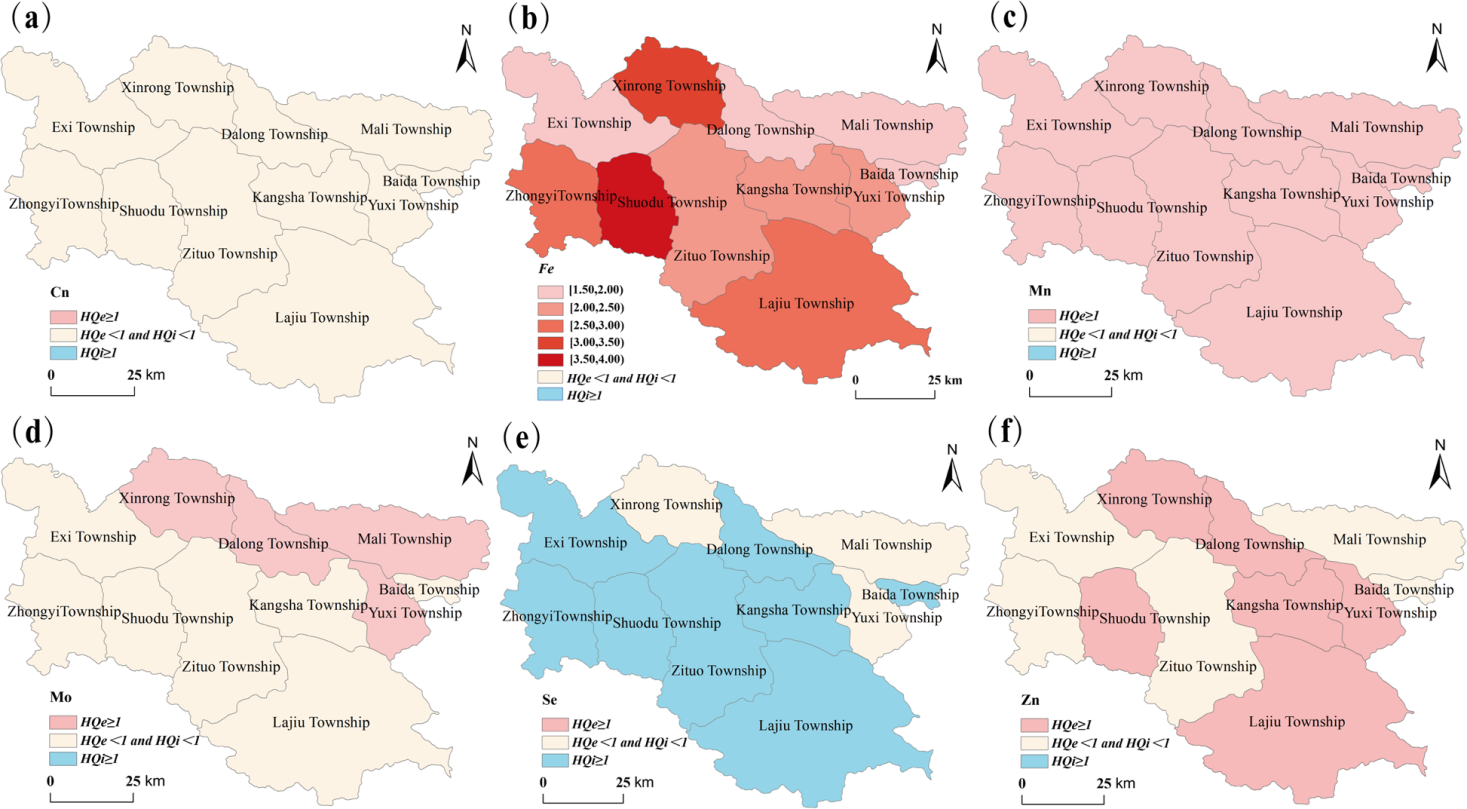
The *HQ* of ETEs in Luolong County, **a** Cu, **b** Fe, **c** Mn, **d** Mo, **e** Se and **f** Zn

The *HQ* of ETEs in each township was calculated according to Eq. (5) and (6). It can be seen from Fig. 3 and Table S7 that the *HQ*_*i*_ and *HQ*_*e*_ values of Cu in each township were less than 1; The *HQ*_*e*_ values of Fe were all greater than 1, ranging from 1.497 (Baida Township) to 3.911 (Shuodu Township); The *HQ*_*e*_ values of Mn were all greater than 1, ranging from 1.2 (Baida Township) to 1.361 (Shuodu Township); The *HQ*_*e*_ values of Mo element in Dalong, Mali, Xinrong and Yuxi townships were slightly greater than 1; the *HQ*_*i*_ values of Se in eight townships were greater than 1; The *HQ*_*e*_ values of Zn in six townships were greater than 1. These data indicate that, there are non-carcinogenic health risks of excessive intake of Fe and Mn in all townships, for non-carcinogenic health risk, 36.36% of townships have health risk of excessive Mo intake, 72.73% of townships have health risk of insufficient Se intake, and 54.55% of townships have health risk of excessive intake of Zn. Therefore, in daily diet, it is necessary to reduce the consumption of food with high Fe, Mn, and Zn concentrations and supply some Se-rich food in order to reduce and control diseases caused by insufficient or excessive intake of ETEs.

## Discussion

The daily diet and drinking water intake of ETEs can directly reflect the health status of local residents [18, 20]. In particular, the relationship between the dietary intake-related diseases and biogeochemistry of ETEs has been the focus of many geochemical and epidemiology studies (seeing the introduction section for review).

Several studies have compared the average concentration of ETEs in drinking water in Tibet or surrounding areas of the study area: Li et al. [52] analyzed the mean concentrations of Fe and Mo in the water of the Qinghai-Tibet Plateau and its surrounding areas, which were 28.75 μg/L and 0.44 μg/L, respectively; LaMu et al. [53] collected and analyzed the average concentrations of Cu, Mn and Zn in surface water of Gongjue County, Qamdo City, which were 0.98 μg/L, 0.73 μg/L and 1.70 μg/L, respectively; Zha et al. [35] sampled and analyzed the concentration of Se in surface water of Tibet, and the average value was 0.52 μg/L. As a result, the concentration of ETEs in drinking water is lower than the average of Tibet or its surrounding areas of the study area, especially Se.

In this study, by comparing the diet and drinking water intakes of ETEs, it was found that residents in Luolong County had a significant excessive *ADD*_*oral*_ intake of Fe, and there was also a slightly excessive intake of Mn, while Mo and Zn were only excessive intaked in some townships (Fig. 2, Table S7). Luolong County belongs to the Bangong Co–Nujiang suture zone, with complex geological structure and strong neotectonic movement [54]. Limonite, pyrite, lead–zinc and gold deposits are widely distributed in the study area, and the main metallogenic elements in the mining area are Fe, Pb, Zn, Ag, Mo, Cu, etc. [55, 56]. These elements can be transported to the hypergene environment through physical and chemical release during weathering, groundwater circulation and ion diffusion [57]. Therefore, the enrichment of Fe, Mn and Zn in the hypergene environment leads to excessive intake of local residents through biogeochemical cycles and food chains.

This study also showed significant deficiencies in oral intake of Se (Table S6). These results demonstrate that KBD patients are concentrated in the southwest of Loulong County, which is exceedingly similar to the distribution pattern of non-carcinogenic health risks of insufficient Se intake, and this finding is consistent with the relationship between the incidence of KBD and biogeochemical Se-deficiency [3, 5]. In addition, according to the preliminary analysis of the risk factors of KBD in Qamdo City and Longzi County by our previous research, the etiology and pathogenesis of KBD are mainly related to the endemic deficiency of some ETEs in local diet [2, 6].

In summary, this study used limited sampling data to assess health risks of residents in Loulong County based on ecosystem supply services, which has reference value for the environmental sustainable development and prevention and control of endemic disease in Loulong County and even Tibet. Firstly, the concentrations of ETEs in local drinking water and highland barley were described and calculated; Secondly, the spatial distribution of ETEs through daily diet *ADD*_*oral*_ were calculated and delineated; Finally, the non-carcinogenic health risks of ETEs in Luolong County were quantitatively analyzed using the health risk assessment methods, and the *HQ* results were visualized, which effectively showed the location of the focus area. However, the samples collected from the study area involved only drinking water and highland barley, which is one-sided when calculating and discussing the *ADD* through dietary intake of ETEs. Moreover, since the average values of element concentrations in each township were used, there were uncertainties in the risk assessment calculations of this study, Therefore, more detailed work needs to be done about health risk assessments in future.

## Conclusion

The concentration of ETEs in daily diet is closely related to the prevalence of KBD and also essential for human health. By calculating the intakes of six major ETEs in diet and drinking water in Luolong County, this research found that there are significant spatial differences in the *ADD*_*oral*_ of ETEs, and it is also confirmed that highland barley is the main oral intake source of ETEs of local residents. The results of this study show that: The oral intake of Cu was appropriate for local residents; The oral intakes of Fe and Mn were excessive and slightly excessive in the whole townships of study area, respectively, which caused a non-carcinogenic health risk to local residents; The townships with non-carcinogenic health risks of oral intake of Mo and Zn accounted for 36.36% and 54.55%, respectively; The *ADD*_*oral*_ of Se was generally low and 72.73% of townships had non-carcinogenic health risks of insufficient Se oral intake. The results also show that there are significant spatial differences in *ADD*_*oral*_ of ETEs, which are closely related to the geographical environment and geological conditions of the study area and human activities. Therefore, it is necessary to reduce the consumption of high concentrations of Fe, Mn and Zn in daily diets and increase the intake of exogenous Mo, Cu, and especially Se-rich food (such as carrot, alfalfa, spinach and corn), which is still the basic countermeasure to prevent and control the prevalence of KBD in local residents.The results of this study also can be helpful to understand the level and distribution of ETEs in the diet of residents in Luolong County, and reduce and control the health risks caused by insufficient or excessive intake of ETEs.

## Supplementary Information

**Additional file 1: Table S1.** The concentration of ETEs in drinking water in Luolong County (unit: μg/L). **Table S2.** The concentration of ETEs in highland barley in Luolong County (unit: mg/kg). **Table S3.** ADDw of ETEs in Luolong County (unit: mg/(kg·day)). **Table S4.** ADDh of ETEs in Luolong County (unit: mg/(kg·day)). **Table S5.** ADDm of ETEs in Luolong County (unit: mg/(kg·day)). **Table S6.** ADDoral of ETEs in Luolong County (unit: mg/(kg·day)). **Table S7.** HQ of ETEs in Luolong County. **Fig. S1.** The map of Distribution of ADDoral in Luolong County. (a) Cu, (b) Fe, (c) Mn, (d) Mo, (e) Se and (f) Zn. **Fig. S2.** The map of distribution of KBD prevalence in Luolong County.

## Data Availability

The data are available for other researchers upon reasonable request.

## Abbreviations

ETEs: Essential trace elements
Cu: Copper
Fe: Iron
Mn: Manganese
Mo: Molybdenum
Se: Selenium
Zn: Zinc
KBD: Kashin-Beck Disease
USEPA: U.S. Environmental Protection Agency
WHO: World Health Organization
FAO: Food and Agriculture Organization of the United Nations
EFSA: European Food Safety Authority
GPS: Global Positioning System
PRA: Participatory rural assessment
MEP: Ministry of Environmental Protection of the People’s Republic of China
NHC: National Health Commission of the People’s Republic of China
℃: Degree centigrade
CAS: Chinese Academy of Sciences
ICP–MS: Inductively Coupled Plasma Mass Spectrometry
HG–AFS: Hydride Generation Atomic Fluorescence Spectrometry
*ADD*: Average daily dose
*C*_*w*_: The mean concentration of trace elements in drinking water
*C*_*h*_: The mean concentration of trace elements in plant food
*C*_*m*_: The mean concentration of trace elements in animal food
*IR*: Ingestion rate
EF: Exposure frequency
ED: Exposure time
BW: Body weight
AT: Average exposure time
RfD: Reference dose
HQ: Hazard quotient
