# Supplementary material for: Dietary and drinking water intake of essential trace elements in a typical Kashin-Beck disease endemic area of Tibet, China
Source: Environ Health. 2022 Sep 16;21:86. doi: 10.1186/s12940-022-00898-0 (PMC9479256; doi:10.1186/s12940-022-00898-0)
Supplement: Supplementary file 1 — Additional file 1: Table S1. The concentration of ETEs in drinking water in Luolong County (unit: μg/L). Table S2. The concentration of ETEs in highland barley in Luolong County (unit: mg/kg). Table S3. ADDw of ETEs in Luolong County (unit: mg/(kg·day)). Table S4. ADDh of ETEs in Luolong County (unit: mg/(kg·day)). Table S5. ADDm of ETEs in Luolong County (unit: mg/(kg·day)). Table S6. ADDoral of ETEs in Luolong County (unit: mg/(kg·day)). Table S7. HQ of ETEs in Luolong County. Fig. S1. The map of Distribution of ADDoral in Luolong County. (a) Cu, (b) Fe, (c) Mn, (d) Mo, (e) Se and (f) Zn. Fig. S2. The map of distribution of KBD prevalence in Luolong County. [file 12940_2022_898_MOESM1_ESM.docx]

**Appendix A.**

**Supplementary** **information**

**Dietary and drinking water intake of essential trace elements in a** **typical Kashin-Beck disease endemic area of Tibet, China**

Xinjie Zha^a,1^, Jialu An^a,1^, Xue Gao^b^, Yuan Tian^c,*^

^a^*Xi'an University of Finance and Economics, Changning Str. 360, Chang'an District, Xi'an, 710100, China*

^b^*Institute of Agricultural Resources and Environment, Tibet Academy of Agriculture and Animal Husbandry Sciences, Jinzhu Str.130, Chengguan District, Lhasa,850000, China*

^c^*Key Laboratory of Ecosystem Network Observation and Modelling, Institute of Geographic Sciences and Natural Resources Research, Chinese Academy of Sciences, Datun Str. 11A, Chaoyang District, Beijing, 100101, China*

**^1^These authors contributed equally to this work.**

**^*^Corresponding author:** Yuan Tian, E-mail: [tiany.16b@igsnrr.ac.cn](mailto:tiany.16b@igsnrr.ac.cn)

# A. Tables

**Table S1**

The concentration of ETEs in drinking water in Luolong County (unit: μg/L)

| Baida Township (n=1) | | | Dalong Township (n=5) | | Exi Township (n=7) | | Kangsha Township (n=14) | |
| --- | --- | --- | --- | --- | --- | --- | --- | --- |
| ETEs | Range | Mean ± SD | Range | Mean ± SD | Range | Mean ± SD | Range | Mean ± SD |
| Cu | 0.189 | 0.189 | 0.142**–**0.394 | 0.897 ± 0.222 | 0.068**–**0.517 | 0.183 ± 0.172 | 0.029**–**0.581 | 0.200 ± 0.176 |
| Fe | 0.370 | 0.370 | 0.621**–**1.156 | 0.040 ± 0.025 | 0.131**–**1.032 | 0.706 ± 0.368 | 0.093**–**2.465 | 0.514 ± 0.744 |
| Mn | 0.041 | 0.041 | 0.019**–**0.075 | 0.041 ± 0.025 | 0.017**–**0.058 | 0.377 ± 0.166 | 0.015**–**0.361 | 0.091 ± 0.107 |
| Mo | 1.252 | 1.252 | 0.314**–**4.319 | 0.198 ± 0.138 | 0.023**–**0.252 | 0.088 ± 0.088 | 0.018**–**0.607 | 0.175 ± 0.164 |
| Se | 0.354 | 0.354 | 0.052**–**0.323 | 0.919 ± 0.701 | 0.031**–**0.197 | 0.088 ± 0.066 | 0.005**–**0.262 | 0.085 ± 0.078 |
| Zn | 0.665 | 0.665 | 0.371**–**1.925 | 0.256 ± 0.100 | 0.177**–**1.027 | 0.614 ± 0.290 | 0.201**–**0.925 | 0.468 ± 0.246 |
| Lajiu Township (n=20) | | | Mali Township (n=7) | | Shuodu Township (n=5) | | Xinrong Township (n=7) | |
| ETEs | Range | Mean ± SD | Range | Mean ± SD | Range | Mean ± SD | Range | Mean ± SD |
| Cu | 0.024**–**0.325 | 0.128 ± 0.091 | 0.125**–**0.391 | 0.206 ± 0.010 | 0.755**–**5.746 | 2.672 ± 2.239 | 0.041**–**0.466 | 0.198 ± 0.146 |
| Fe | 0.082**–**2.733 | 0.688 ± 0.647 | 0.165**–**0.657 | 0.384 ± 0.206 | 0.081**–**0.409 | 0.264 ± 0.139 | 0.091**–**2.218 | 0.963 ± 0.825 |
| Mn | 0.004**–**0.088 | 0.034 ± 0.023 | 0.016**–**0.079 | 0.053 ± 0.024 | 0.027**–**4.012 | 1.370 ± 2.288 | 0.007**–**0.205 | 0.075 ± 0.071 |
| Mo | 0.009**–**2.123 | 0.652 ± 0.628 | 0.092**–**0.938 | 0.432 ± 0.354 | 0.011**–**0.666 | 0.219 ± 0.286 | 0.021**–**0.202 | 0.105 ± 0.073 |
| Se | 0.008**–**0.087 | 0.035 ± 0.023 | 0.148**–**0.483 | 0.303 ± 0.130 | 0.024**–**0.175 | 0.074 ± 0.066 | 0.046**–**0.788 | 0.336 ± 0.295 |
| Zn | 0.120**–**0.844 | 0.392 ± 0.196 | 0.162**–**2.510 | 0.932 ± 0.961 | 0.200**–**5.358 | 1.965 ± 2.135 | 0.367**–**1.602 | 0.850 ± 0.431 |

**Continued Table S1**

| Yuxi Township (n=5) | | | Zhongyi Township (n=6) | | Zituo Township (n=12) | | Luolong County (n=89) | |
| --- | --- | --- | --- | --- | --- | --- | --- | --- |
| ETEs | Range | Mean ± SD | Range | Mean ± SD | Range | Mean ± SD | Range | Mean ± SD |
| Cu | 0.048**–**0.244 | 0.141 ± 0.074 | 0.489**–**1.247 | 0.747 ± 0.279 | 0.042**–**0.628 | 0.270 ± 0.192 | 0.024**–**1.360 | 0.278 ± 0.264 |
| Fe | 0.099**–**0.311 | 0.215 ± 0.088 | 0.708**–**1.381 | 1.070 ± 0.287 | 0.278**–**25.074 | 4.871 ± 8.230 | 0.081**–**25.074 | 0.766 ± 0.312 |
| Mn | 0.022**–**0.131 | 0.058 ± 0.050 | 0.039**–**1.432 | 0.452 ± 0.591 | 0.023**–**0.307 | 0.075 ± 0.088 | 0.004**–**1.432 | 0.119 ± 0.223 |
| Mo | 0.180**–**0.365 | 0.273 ± 0.070 | 0.014**–**0.201 | 0.083 ± 0.078 | 0.017**–**2.327 | 0.491 ± 0.658 | 0.009**–**2.151 | 0.411 ± 0.526 |
| Se | 0.158**–**0.538 | 0.308 ± 0.147 | 0.023**–**0.539 | 0.149 ± 0.207 | 0.007**–**0.170 | 0.066 ± 0.049 | 0.005**–**0.788 | 0.155 ± 0.180 |
| Zn | 0.171**–**1.274 | 0.560 ± 0.501 | 0.092**–**6.950 | 2.114 ± 2.779 | 0.211**–**13.540 | 3.311 ± 4.777 | 0.092**–**13.540 | 0.804 ± 1.112 |

**Table S2**

The concentration of ETEs in highland barley in Luolong County (unit: mg/kg)

| Baida Township (n=2) | | | Dalong Township (n=3) | | Exi Township (n=8) | | Kangsha Township (n=9) | |
| --- | --- | --- | --- | --- | --- | --- | --- | --- |
| ETEs | Range | Mean ± SD | Range | Mean ± SD | Range | Mean ± SD | Range | Mean ± SD |
| Cu | 3.709**–**4.085 | 3.897 ± 0.266 | 3.117**–**5.269 | 4.251 ± 1.081 | 3.180**–**5.350 | 3.896 ± 0.732 | 3.253**–**4.883 | 4.166 ± 0.650 |
| Fe | 35.31**–**40.35 | 37.83 ± 3.56 | 35.40**–**67.74 | 51.20 ± 16.19 | 41.58**–**77.58 | 52.53 ± 13.24 | 44.12**–**123.60 | 76.84 ± 25.168 |
| Mn | 13.03**–**13.36 | 13.20 ± 0.23 | 13.24**–**13.87 | 13.49 ± 0.34 | 13.12**–**14.70 | 13.75 ± 0.69 | 11.71**–**14.86 | 13.55 ± 1.36 |
| Mo | 0.508**–**0.510 | 0.509 ± 0.001 | 0.486**–**1.132 | 0.728 ± 0.352 | 0.048**–**0.432 | 0.217 ± 0.134 | 0.234**–**1.108 | 0.615 ± 0.371 |
| Se | 0.0033**–**0.0059 | 0.0046 ± 0.0020 | 0.0008**–**0.0041 | 0.0020 ± 0.0020 | 0.0020**–**0.0030 | 0.0024 ± 0.0005 | 0.0016**–**0.0040 | 0.0030 ± 0.0008 |
| Zn | 19.54**–**22.87 | 21.21 ± 2.36 | 24.92**–**28.6 | 27.13 ± 1.95 | 17.87**–**26.93 | 22.91 ± 2.90 | 22.19**–**31.43 | 25.86 ± 3.97 |
| Lajiu Township (n=15) | | | Mali Township (n=6) | | Shuodu Township (n=15) | | Xinrong Township (n=3) | |
| ETEs | Range | Mean ± SD | Range | Mean ± SD | Range | Mean ± SD | Range | Mean ± SD |
| Cu | 2.751**–**4.542 | 3.424 ± 0.542 | 3.591**–**5.612 | 4.443 ± 0.834 | 1.974**–**4.052 | 3.129 ± 0.510 | 3.301**–**4.203 | 3.660 ± 0.479 |
| Fe | 51.46**–**135.00 | 84.86 ± 25.23 | 36.22**–**128.50 | 58.55 ± 34.90 | 42.99**–**250.73 | 139.64 ± 70.12 | 53.61**–**143.90 | 107.74 ± 47.75 |
| Mn | 11.99**–**16.25 | 14.099 ± 1.66 | 11.43**–**15.61 | 13.988 ± 1.826 | 13.13**–**16.761 | 14.966 ± 1.003 | 13.23**–**13.99 | 13.56 ± 0.39 |
| Mo | 0.152**–**0.772 | 0.404 ± 0.212 | 0.380**–**1.115 | 0.728 ± 0.244 | 0.095**–**0.507 | 0.228 ± 0.109 | 0.285**–**0.781 | 0.566 ± 0.254 |
| Se | 0.0003**–**0.0034 | 0.0020 ± 0.0010 | 0.0059**–**0.0150 | 0.0100 ± 0.0040 | 0.0013**–**0.0029 | 0.0020 ± 0.0004 | 0.0018**–**0.0220 | 0.0090 ± 0.0110 |
| Zn | 16.71**–**27.77 | 23.74 ± 2.95 | 14.67**–**28.94 | 22.35 ± 5.95 | 21.68**–**28.06 | 24.63 ± 1.94 | 22.26**–**25.85 | 23.82 ± 1.84 |

**Continued Table S2**

| Yuxi Township (n=2) | | | Zhongyi Township (n=4) | | Zituo Township (n=18) | | Luolong County (n=85) | |
| --- | --- | --- | --- | --- | --- | --- | --- | --- |
| ETEs | Range | Mean ± SD | Range | Mean ± SD | Range | Mean ± SD | Range | Mean ± SD |
| Cu | 4.197**–**5.273 | 4.740 ± 0.760 | 2.777**–**3.934 | 3.223 ± 0.503 | 2.331**–**3.957 | 3.220 ± 0.454 | 1.970-5.270 | 3.550 ± 0.680 |
| Fe | 58.67**–**62.29 | 60.48 ± 2.56 | 54.48**–**125.66 | 97.74 ± 33.76 | 37.35**–**167.70 | 79.22 ± 42.37 | 35.31**–**182.30 | 81.17 ± 38.14 |
| Mn | 12.43**–**16.45 | 14.44 ± 2.84 | 10.61**–**14.95 | 13.50 ± 1.96 | 11.00**–**16.17 | 13.64 ± 1.30 | 10.61**–**16.79 | 14.03 ± 1.42 |
| Mo | 0.603**–**0.720 | 0.661 ± 0.082 | 0.295**–**0.442 | 0.353 ± 0.066 | 0.012**–**0.416 | 0.228 ± 0.120 | 0.010**–**0.840 | 0.350 ± 0.200 |
| Se | 0.0130**–**0.0290 | 0.0210 ± 0.0110 | 0.0018**–**0.0046 | 0.0030 ± 0.0010 | 0.0008**–**0.0024 | 0.0010 ± 0.0004 | 0.0003**–**0.0502 | 0.0028 ± 0.0056 |
| Zn | 21.91**–**33.74 | 27.83 ± 8.37 | 19.47**–**27.49 | 23.61 ± 3.36 | 19.47**–**24.5 | 21.83 ± 1.21 | 14.67**–**31.43 | 23.58 ± 3.10 |

**Table S3**

*ADD_w_* of ETEs in Luolong County (unit: mg/(kg·day))

| ETEs | Baida | Dalong | Exi | Kangsha | Lajiu | Mali | Shuodu | Xinrong | Yuxi | Zhongyi | Zituo |
| --- | --- | --- | --- | --- | --- | --- | --- | --- | --- | --- | --- |
| Cu | 1.22E-05 | 1.65E-05 | 1.18E-05 | 1.29E-05 | 5.61E-07 | 1.33E-05 | 1.72E-04 | 1.28E-05 | 9.12E-06 | 4.82E-05 | 1.74E-05 |
| Fe | 2.39E-05 | 5.79E-05 | 4.55E-05 | 3.32E-05 | 4.44E-05 | 2.48E-05 | 1.70E-05 | 6.21E-05 | 1.38E-05 | 6.90E-05 | 3.14E-04 |
| Mn | 2.65E-06 | 2.58E-06 | 2.43E-06 | 5.90E-06 | 2.18E-06 | 3.46E-06 | 8.84E-05 | 4.83E-06 | 3.74E-06 | 2.92E-05 | 4.81E-06 |
| Mo | 8.08E-05 | 1.09E-04 | 5.65E-06 | 1.13E-05 | 2.24E-06 | 2.79E-05 | 1.42E-05 | 6.75E-06 | 1.76E-05 | 5.33E-06 | 3.17E-05 |
| Se | 2.28E-05 | 1.28E-05 | 5.65E-06 | 5.51E-06 | 2.53E-05 | 1.96E-05 | 4.75E-06 | 2.17E-05 | 1.99E-05 | 9.59E-06 | 4.24E-06 |
| Zn | 4.29E-05 | 5.93E-05 | 3.96E-05 | 3.02E-05 | 8.28E-06 | 6.01E-05 | 1.27E-04 | 5.49E-05 | 3.61E-05 | 1.36E-04 | 2.14E-04 |

**Table S4**

*ADD_h_* of ETEs in Luolong County (unit: mg/(kg·day))

| ETEs | Baida | Dalong | Exi | Kangsha | Lajiu | Mali | Shuodu | Xinrong | Yuxi | Zhongyi | Zituo |
| --- | --- | --- | --- | --- | --- | --- | --- | --- | --- | --- | --- |
| Cu | 0.0727 | 0.0740 | 0.0689 | 0.0725 | 0.0546 | 0.0802 | 0.0600 | 0.0677 | 0.0911 | 0.0610 | 0.0597 |
| Fe | 0.6814 | 0.9204 | 0.9461 | 1.3839 | 1.5285 | 0.8026 | 2.5150 | 1.9404 | 1.0893 | 1.7604 | 1.4268 |
| Mn | 0.2377 | 0.2429 | 0.2477 | 0.2440 | 0.2539 | 0.2594 | 0.2695 | 0.2443 | 0.2601 | 0.2431 | 0.2457 |
| Mo | 0.0107 | 0.0161 | 0.0054 | 0.0113 | 0.0093 | 0.0141 | 0.0042 | 0.0146 | 0.0143 | 0.0062 | 0.0057 |
| Se | 8.29E-05 | 3.57E-05 | 4.36E-05 | 5.84E-05 | 3.00E-05 | 1.88E-04 | 3.69E-05 | 1.58E-04 | 3.78E-04 | 5.04E-05 | 2.26E-05 |
| Zn | 0.3819 | 0.4886 | 0.4127 | 0.4658 | 0.4276 | 0.4025 | 0.4436 | 0.4291 | 0.5012 | 0.4251 | 0.3932 |

**Table S5**

*ADD_m_* of ETEs in Luolong County (unit: mg/(kg·day))

| ETEs | Cu(n=15) | Fe(n=15) | Mn(n=15) | Mo(n=8) | Se(n=15) | Zn(n=15) |
| --- | --- | --- | --- | --- | --- | --- |
| ADD | 0.0112 | 0.2223 | 0.0021 | 0.0025 | 0.0008 | 0.0862 |

**Note : The average content data of essential trace elements in meat are from(Wu, 2017) and (Wu, 2020).**

**Table S6**

*ADD_oral_* of ETEs in Luolong County (unit: mg/(kg·day))

| ETEs | Baida | Dalong | Exi | Kangsha | Lajiu | Mali | Shuodu | Xinrong | Yuxi | Zhongyi | Zituo |
| --- | --- | --- | --- | --- | --- | --- | --- | --- | --- | --- | --- |
| Cu | 0.08099 | 0.08232 | 0.07722 | 0.08081 | 0.06292 | 0.08855 | 0.06839 | 0.07602 | 0.09941 | 0.06933 | 0.06805 |
| Fe | 1.13665 | 1.37567 | 1.40144 | 1.83920 | 1.98377 | 1.25793 | 2.97026 | 2.39575 | 1.54459 | 2.21574 | 1.88225 |
| Mn | 0.23874 | 0.24399 | 0.24878 | 0.24509 | 0.25503 | 0.26053 | 0.27068 | 0.24537 | 0.26116 | 0.24419 | 0.24683 |
| Mo | 0.01333 | 0.01874 | 0.00793 | 0.01381 | 0.01184 | 0.01668 | 0.00671 | 0.01716 | 0.01684 | 0.00876 | 0.00827 |
| Se | 0.00086 | 0.00080 | 0.00081 | 0.00082 | 0.00079 | 0.00096 | 0.00080 | 0.00093 | 0.00115 | 0.00082 | 0.00079 |
| Zn | 0.67945 | 0.78612 | 0.71021 | 0.76332 | 0.72512 | 0.70000 | 0.74119 | 0.72662 | 0.79868 | 0.72273 | 0.69083 |

**Table S7**

*HQ* of ETEs in Luolong County

| ETEs | Cu | | Fe | | Mn | | Mo | | Se | | Zn | |
| --- | --- | --- | --- | --- | --- | --- | --- | --- | --- | --- | --- | --- |
| *HQ* | *HQ_i_* | *HQ_e_* | *HQ_i_* | *HQ_e_* | *HQ_i_* | *HQ_e_* | *HQ_i_* | *HQ_e_* | *HQ_i_* | *HQ_e_* | *HQ_i_* | *HQ_e_* |
| Baida | 0.133 | 0.560 | 0.191 | 1.497 | 0.311 | 1.200 | 0.113 | 0.818 | 1.050 | 0.119 | 0.220 | 0.939 |
| Dalong | 0.131 | 0.569 | 0.158 | 1.811 | 0.305 | 1.227 | 0.080 | 1.150 | 1.119 | 0.112 | 0.190 | 1.087 |
| Exi | 0.140 | 0.534 | 0.155 | 1.845 | 0.299 | 1.251 | 0.189 | 0.487 | 1.113 | 0.112 | 0.210 | 0.982 |
| Kangsha | 0.134 | 0.558 | 0.118 | 2.422 | 0.303 | 1.232 | 0.109 | 0.847 | 1.093 | 0.114 | 0.195 | 1.055 |
| Lajiu | 0.172 | 0.435 | 0.109 | 2.612 | 0.291 | 1.282 | 0.127 | 0.726 | 1.132 | 0.110 | 0.206 | 1.003 |
| Mali | 0.122 | 0.612 | 0.173 | 1.656 | 0.285 | 1.310 | 0.090 | 1.023 | 0.937 | 0.133 | 0.213 | 0.968 |
| Shuodu | 0.158 | 0.473 | 0.073 | 3.911 | 0.274 | 1.361 | 0.224 | 0.412 | 1.123 | 0.111 | 0.201 | 1.025 |
| Xinrong | 0.142 | 0.525 | 0.091 | 3.154 | 0.303 | 1.234 | 0.087 | 1.053 | 0.966 | 0.129 | 0.205 | 1.005 |
| Yuxi | 0.109 | 0.687 | 0.140 | 2.034 | 0.284 | 1.313 | 0.089 | 1.033 | 0.782 | 0.160 | 0.187 | 1.104 |
| Zhongyi | 0.156 | 0.479 | 0.098 | 2.917 | 0.304 | 1.228 | 0.171 | 0.537 | 1.101 | 0.114 | 0.206 | 0.999 |
| Zituo | 0.159 | 0.470 | 0.115 | 2.478 | 0.301 | 1.241 | 0.181 | 0.507 | 1.144 | 0.109 | 0.216 | 0.955 |
| Luolong | 0.150 | 0.499 | 0.113 | 2.524 | 0.293 | 1.276 | 0.171 | 0.539 | 1.094 | 0.114 | 0.207 | 0.999 |

# B. Figures


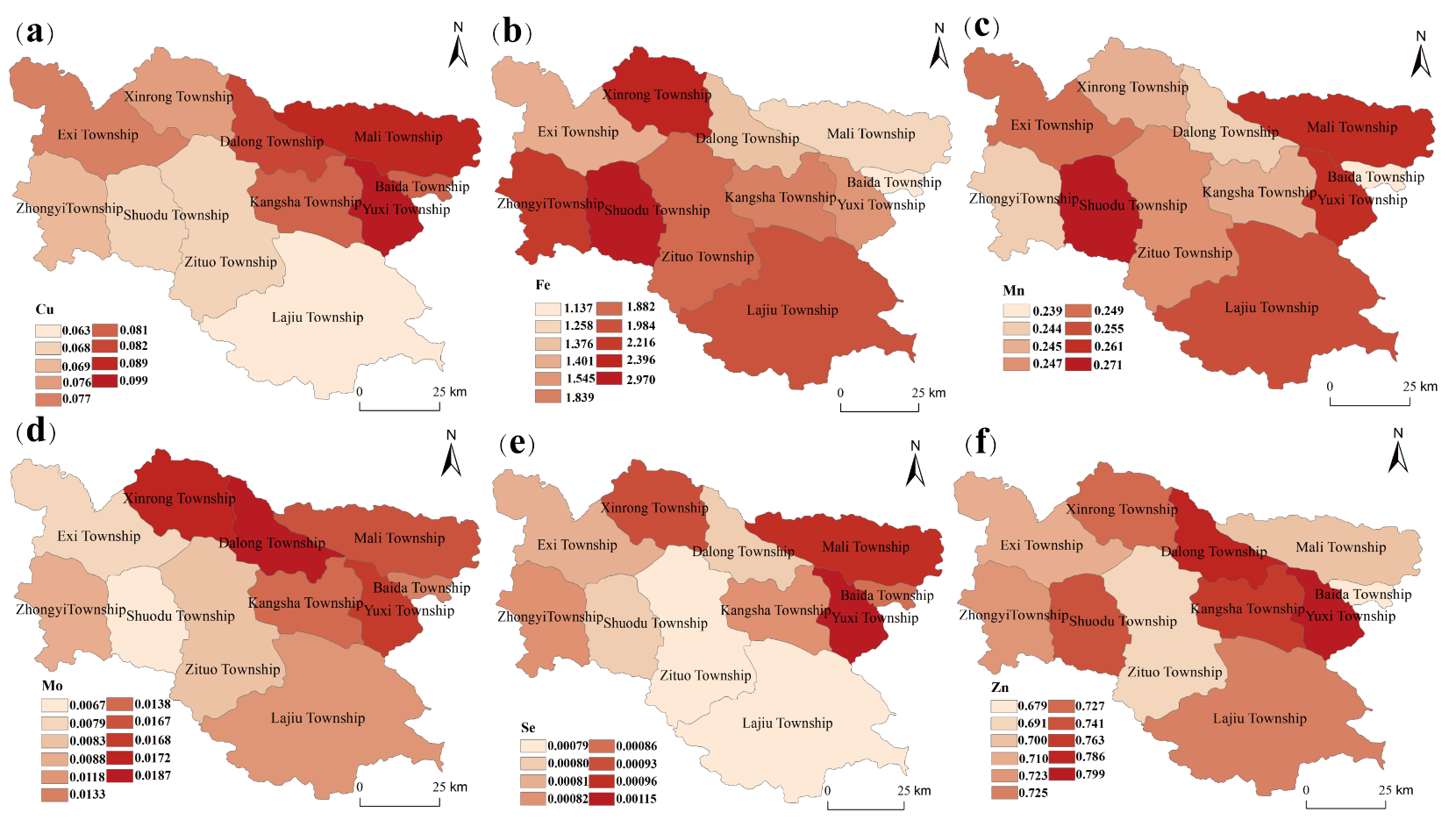


**Fig. S1.** The map of Distribution of *ADD_oral_* in Luolong County. (a) Cu, (b) Fe, (c) Mn, (d) Mo, (e) Se and (f) Zn


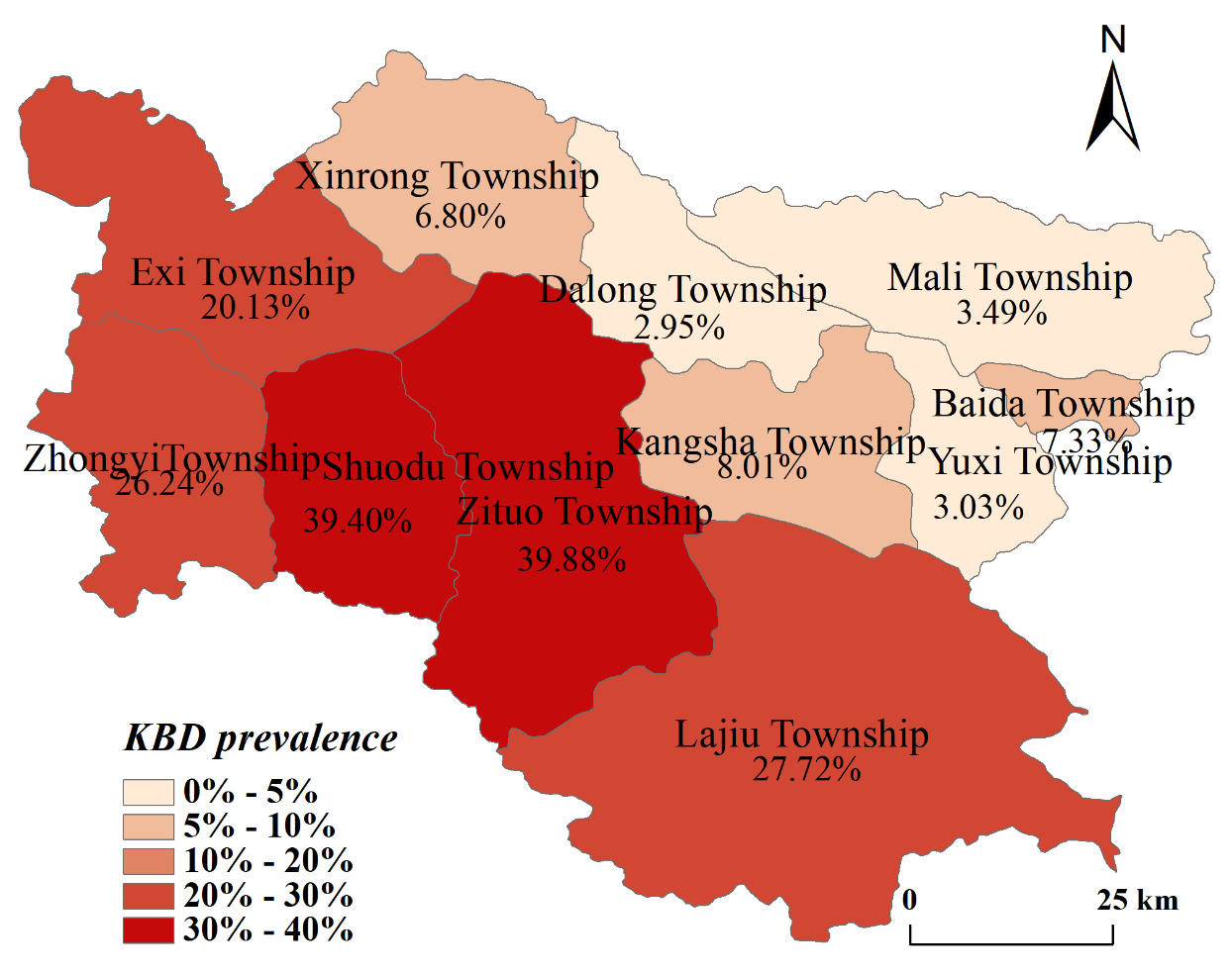


**Fig. S2.** The map of distribution of KBD prevalence in Luolong County.

# C. Reference

Wu X. Determination of trace elements in Tibet yak meat by inductively coupled plasma atomic emission spectrometry. Tibet Journal of Agricultural Sciences. 2017; 39(3): 29-33.

Wu X. Correlation analysis of mineral elements in yak meat and Herbage in seven regions of Tibet. Tibet Journal of Agricultural Sciences. 2020; 42(4):14-7.
